# Brain vital sign monitoring of sleep deprivation detects situational cognitive impairment

**DOI:** 10.3389/fnhum.2024.1358551

**Published:** 2024-04-02

**Authors:** Katherine B. Jones, Tory Frizzell, Shaun Fickling, Gabriela Pawlowski, Sonia M. Brodie, Bimal Lakhani, Jan Venter, Ryan C. N. D’Arcy

**Affiliations:** ^1^Centre for Neurology Studies, HealthTech Connex, Vancouver, BC, Canada; ^2^BrainNET, Health and Technology District, Surrey, BC, Canada; ^3^Healthcode Ltd, Vancouver, BC, Canada; ^4^Faculty of Applied Sciences, Simon Fraser University, Burnaby, BC, Canada

**Keywords:** sleep deprivation, brain vital signs, cognitive impairment, ERP, clinical neuroscience

## Abstract

Objective, rapid evaluation of cognitive function is critical for identifying situational impairment due to sleep deprivation. The present study used brain vital sign monitoring to evaluate acute changes in cognitive function for healthy adults. Thirty (30) participants were scanned using portable electroencephalography before and after either a night of regular sleep or a night of total sleep deprivation. Brain vital signs were extracted from three established event-related potential components: (1) the N100 (Auditory sensation); (2) the P300 (Basic attention); and (3) the N400 (Cognitive processing) for all time points. As predicted, the P300 amplitude was significantly reduced in the sleep deprivation group. The findings indicate that it is possible to detect situational cognitive impairment due to sleep deprivation using objective, rapid brain vital sign monitoring.

## 1 Introduction

Cognitive impairment is often associated with a wide range of conditions, including Alzheimer’s Disease, traumatic brain injury, Parkinson’s Disease, schizophrenia, epilepsy, and stroke (Petersen, 2007; Jackson et al., 2014; Nandrajog et al., 2017; Daniel Slater et al., 2019; Zhong et al., 2019). However, cognitive impairment can also occur in otherwise healthy individuals during situations that adversely affect information processing (i.e., situational cognitive impairment). Sleep deprivation is among the most common causes of situational cognitive impairment. Globally, 62% of adults report poor sleep (The Global Pursuit of Better Sleep Health, 2019), and in the US alone, more than 1 in 3 individuals are sleep deprived (Liu et al., 2019). The ability to assess cognitive function efficiently and objectively is critical for early and sensitive detection of cognitive impairment–important for real world performance and safety issues related to sleep deprivation and for the development of effective strategies for intervention.

Previous research has suggested that cognitive function can be objectively evaluated using event-related potentials (ERPs) (Connolly et al., 2000; Gawryluk et al., 2010; Zhong et al., 2019; Lima et al., 2022). Extracted from electroencephalography (EEG), ERPs reflect the brain’s non-invasive response during sensory, attention, and cognitive processing (Luck, 2014). Specifically, the ERP components of interest include: the N100, which serves as a gauge of auditory sensation and has been studied since 1939 (Davis, 1939; Näätänen and Picton, 1987), the P300, that reflects the brain response in attention paradigms and is one of the most studied ERP across a large range of conditions (Sutton et al., 1967; Patel and Azzam, 2005; Polich, 2007), and the N400 response, that has also been extensively explored in neurological conditions like dyslexia, stroke, traumatic brain injury, and dementia (D’Arcy et al., 2003; Taylor and Olichney, 2007; Schulz et al., 2008; Steppacher et al., 2013; Ghosh Hajra et al., 2018). The N400 ERP response reflects cognitive language processing, particularly the brain’s response to unexpected semantic incongruity.

As such, certain ERPs are sensitive to changes in cognitive processing related to sleep deprivation. In particular, numerous studies have shown that changes in the P300, an ERP component related to attention and information processing, is affected by sleep deprivation (Morris et al., 1992; Lee et al., 2003; Kusztor et al., 2019; Zhang et al., 2019; Lima et al., 2022). In contrast, the N100 reflects lower-level sensory processing and is consequently relatively unaffected by sleep deprivation (Lee et al., 2004; Jackson et al., 2008). Limited research has investigated the effect of sleep deprivation on the N400, which is sensitive to cognitive semantic processing (Ito, 1997; Ghosh Hajra et al., 2018; Ledwidge et al., 2022). López Zunini et al. (2014) found a reduction in N400 amplitude in response to total sleep deprivation, while Tavakoli et al. (2015) found no effect of partial sleep deprivation on the N400.

In 2016, we developed a brain vital sign framework that incorporates the N100, P300, and N400 into a rapid, standardized, intuitive, and easy to use approach to evaluate cognitive brain function (Ghosh Hajra et al., 2016). The brain vital sign framework has since been validated increasingly in both health individuals as well as individuals with brain injury (including concussion) and brain disease (Ghosh Hajra et al., 2018; Fickling et al., 2019b,Smith et al., 2020; Carrick et al., 2021; Fickling et al., 2021; Kirby et al., 2023). Sleep deprivation can lead to a wide decline across domains of cognitive performance, such as response speed, attention, memory, verbal comprehension, mood, et cetera (Pilcher and Huffcutt, 1996; Kim et al., 2001; Philibert, 2005; Walker, 2008). In this instance, both the P300 (basic attention) and the N400 (cognitive processing) responses represent potential indicators for cognitive impairment. If validated, the rapid and portable translation of brain vital signs enables a wide array of evaluations beyond the laboratory environment, where sleep deprivation and situational cognitive impairment is a key concern.

In the present study, brain vital sign evaluations were conducted using the NeuroCatch Platform. NeuroCatch uses an auditory stimulus sequence including auditory oddball stimuli and word pairs to elicit the N100, P300, and N400 responses from a rapid EEG scan. In this study, 30 participants were assessed before and after either a typical night of sleep or a night of total sleep deprivation. The study objective was to evaluate whether situational cognitive impairment, due to sleep deprivation, could be detected through monitoring brain vital signs. The hypothesis predicted specific sleep deprivation impacts on the P300 and the N400.

## 2 Materials and methods

### 2.1 Participants

Participants were healthy adults with self-described regular sleep patterns for at least the previous 2 weeks, no diagnosis of any sleep disorder, and not currently taking sleep medications, supplements, or medications that effect sleep (see Supplementary material for full list of criteria).

A pilot study was completed with 3 participants to assess required sample size. These data were not included in the study analysis. Sample size was calculated using GPower (version 3.1.9.7) based on estimated moderate effect sizes for the primary repeated measures analysis.

Effect Size: 0.25, Alpha Error Probability: 0.00833 (Bonferonni’s Adjusted for multiple comparisons) Beta Error Probability: 0.8 Groups: 2 Measurements: 2 Correlation among repeated measures: 0.8.

In addition, 0.8 is the estimated minimum test–retest correlation coefficient reported by Trejo et al. (1991) in a study of auditory evoked potentials using rare and frequent tones.

The minimum required sample size was determined to be 30 participants, and accounting for a 20% contingency for participant dropout the total minimum intended screening was 36 participants.

In total, 38 participants were screened, of which 37 passed screening. Five participants who passed screening were unable to participate due to schedule conflicts or COVID-19 related issues. The remaining 32 participants were randomly assigned to either the Sleep Deprivation (SDEP) or Control Group (CTRL). Two participants withdrew from the study prior to the Day 2 assessments. All remaining participants completed the study in groups. There was no significant difference in age between the SDEP and CTRL groups [*t*(28) = 1.08, *p* = 0.289]. See Table 1 for descriptive statistics of the sample.

**TABLE 1:** Participant demographics.

Group	*N*	Age	Sex	Handedness
SDEP	15	31.07 ± 6.32	8 female, 7 male	14 right, 1 left
CTRL	15	28.60 ± 5.75	10 female, 5 male	13 right, 1 left, 1 both

### 2.2 Procedures

This study was approved by the Advarra Canadian Research Ethics Board and registered on ClinicalTrials.gov (NCT05560620). The study was a randomized, interventional, mixed-design study that included two groups: SDEP (*n* = 15) and CTRL (*n* = 15). A total of 3 sessions were conducted, including 8-12 participants each. All participants completed assessments at 3 primary timepoints, split across 2 consecutive days: Day 1 morning (Baseline 1, ∼8:30–10:00 a.m.), Day 1 evening (Baseline 2, ∼8:00–9:30 p.m.), Day 2 morning (Post 1, ∼8:30–10:00 a.m.). After Baseline 2, CTRL participants left the lab to follow their regular sleep routine and returned for Day 2 assessments. SDEP participants stayed in the lab and remained awake overnight through the Day 2 assessments, which ended at approximately 12 p.m.

A controlled caffeine ingestion intervention was completed after the described study (Post 2, ∼10:30–12:00 p.m.), and has been analyzed as a separate study.

Overnight, the SDEP participants stayed in a large conference room, with two research staff present. A variety of activities were available to the participants such as games, movies, puzzles, and coloring books. Participants were provided with food and water throughout the night. From Baseline 2 through to Post 1 assessment, SDEP participants did not consume caffeine.

### 2.3 Measures

Outcome measures at each of the timepoints included NeuroCatch scans and additional behavioral cognitive assessments. The present paper will focus on the NeuroCatch scan results specific to brain vital sign monitoring. Behavioral cognitive assessment results are the focus of separate analyses.

#### 2.3.1 EEG scan

NeuroCatch (HealthTech Connex Inc, BC, Canada) is a rapid, portable, and standardized evaluation of brain vital signs as markers of cognitive brain function. A pre-scan, digital survey consists of questions regarding the participants’ self-described mood, total sleep (hours), caffeine intake, alcohol consumption, nicotine usage, psychoactive usage, and medication usage over the last 24 h. Following the survey, a low-density EEG sensor cap (ANT Neuro Waveguard) with standard Ag/AgCl electrodes was fitted to the participants, and skin-electrode impedances were prepared to below 25 KOhms. Data were recorded from 3 midline electrodes (Fz, Cz, and Pz), with a ground electrode located at Afz, a reference electrode placed on the left earlobe, and a single electrooculogram (EOG) recorded from FPz. The scan takes approximately 6 min and involves repeated auditory stimulation (ear insert headphones) of standard (80 dB) and random rare deviant (105 dB) tone trains ahead of basic spoken word pair primes that either match or mismatch (e.g., pizza/cheese, pizza/window, respectively). The N100 and P300 ERP peaks were identified on the auditory oddball stimuli response (i.e., the rare deviant tones) and the N400 ERP peaks were identified on the semantic mismatch word response. An additional scan was also acquired if the first scan was identified with poor signal quality. During the scans, participants were asked to sit still and fixate on a cross positioned eye-level ∼2 m away. Scans were conducted in quiet, closed rooms to reduce visual and auditory distractions.

#### 2.3.2 Pre-processing

Recorded EEG traces were processed in Python. EEG were filtered using a 0.1–20 Hz bandpass and 60 Hz notch filter. Ocular artifacts were corrected using an adaptive filter (He et al., 2004) with the EOG derived from the FPz channel. Stimulus-locked evoked epochs were extracted according to stimulus condition (i.e., standard/deviant tones, congruent/incongruent words). Epochs containing artifacts were rejected using an automated EEG signal-quality index (Fickling et al., 2019a). Artifact-free epochs were averaged for each stimulus condition to form representative ERP waveforms for each participant. Grand-average ERPs were then derived for each group by averaging the ERPs for the relevant participants. A non-parametric resampling method (di Nocera and Ferlazzo, 2000) was used to derive 90% confidence intervals of the grand-average waveforms for visualization. ERP peaks were identified by a blinded expert and verified by a second reviewer. Discrepancies were corrected by 3rd reviewer if a tiebreaker was required. Peaks were identified on the waveforms for each scan as a single amplitude value at a chosen latency.

### 2.4 Statistical analysis

Statistical analysis was conducted in SPSS (IBM SPSS Statistics 29.0.1.0). A mass-univariate repeated measures analysis of variance (Groppe et al., 2011a,b) was conducted at the group waveform level to test the null hypothesis that there was no differential effect of time on the evoked responses between the two groups.

## 3 Results

The mass univariate two-way repeated measures ANOVA indicated a significant interaction between group and changes in the P300 amplitude over time [*F*_(2,1)_ = 3.25, *p* = 0.047, η^2^ = 0.115]. Breaking down this interaction revealed a significant difference between T2 (Day 1 evening) and T3 (Day 2 morning) in the sleep deprivation group only (*p* = 0.028), such that following a night of sleep deprivation, the P300 amplitude was significantly reduced.

An overall significant effect of time on N400 amplitude was observed [*F*_(2,1)_ = 4.45, *p* = 0.017, η^2^ = 0.151], with amplitude decreasing over time. Specifically, a significant reduction in N400 amplitude was observed between T1 (Day 1 morning) and T3 (Day 2 morning) (*p* = 0.031). No significant effect of time, or time by group interaction was observed for the N100 amplitude or latency, or for the latencies for the P300 or N400.

**FIGURE 1:**
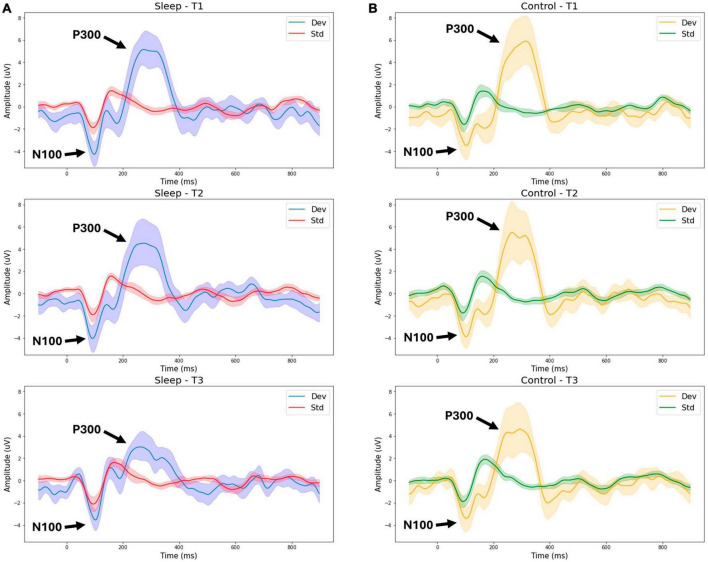
Group average waveforms for the P300 (tone stimuli). T1—Day 1 Morning (Baseline 1). T2—Day 1 Evening (Baseline 2). T3—Day 2 Morning (Post 1). Sleep—sleep deprivation group **(A)** or Control—normal sleep **(B)**. Dev—deviant tone response, Std—standard tone response.

**FIGURE 2:**
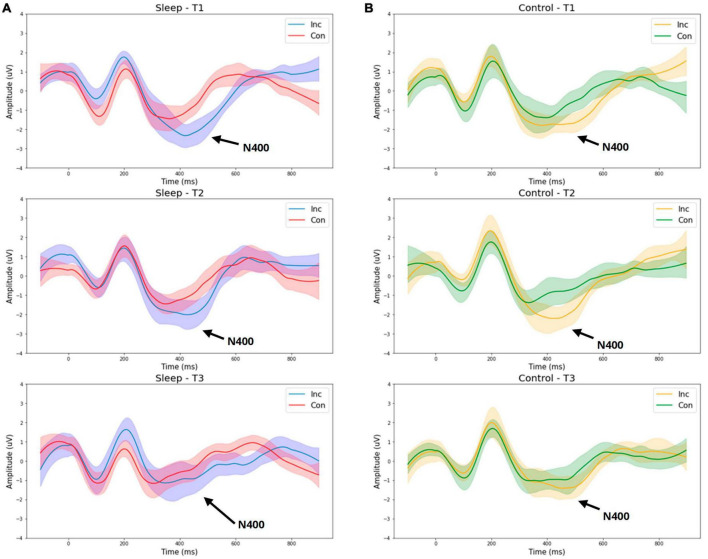
Group average waveforms for the N400 (word pair stimuli). T1—Day 1 Morning (Baseline 1). T2—Day 1 Evening (Baseline 2). T3—Day 2 Morning (Post 1). Sleep –sleep deprivation group **(A)** or Control—normal sleep **(B)**. Inc—Incongruent word response, Con—Congruent word response.

Figure 1 presents grand average waveforms for the P300 response to standard and deviant tones for the SDEP and CTRL groups across timepoints 1, 2, and 3 (Day 1 morning, Day 1 evening, Day 2 morning, respectively). Figure 2 presents grand average waveforms for the N400 response to congruent and incongruent words for the SDEP and CTRL groups across timepoints 1, 2, and 3. The 95% confidence intervals of the mean for each waveform are shaded.

## 4 Discussion

### 4.1 Main findings

With the objective to evaluate situational cognitive impairment due to sleep deprivation, the findings supported the hypothesis that brain vital signs would show sleep deprivation effects (Figure 1). Specifically, a significant reduction in P300 amplitude between T2 (Day 1 evening) and T3 (Day 2 morning) was observed in the sleep deprivation group only. This reduction of the P300 amplitude following sleep deprivation reduction is strongly consistent with prior research (Lee et al., 2003; Kusztor et al., 2019; Lima et al., 2022).

While no significant interaction between time and group was observed for the N400 amplitude, examination of the waveforms suggests the sleep deprivation group overall exhibited a greater reduction in N400 amplitude from T2 (day 1 evening) to T3 (day 2 morning) compared with the control group (Figure 2). Possibly, with a larger sample size, an interaction between time and group on the N400 amplitude would have been detected, consistent with prior research demonstrating reduced N400 amplitude following total sleep deprivation (López Zunini et al., 2014).

Regarding the N100 component, there was no significant change observed between groups. This pattern of results is consistent with prior literature (Lee et al., 2004; Jackson et al., 2008), and understandable given the exogenous nature of the N100 response (Lee et al., 2004; Jackson et al., 2008; Joos et al., 2014). Additionally, no significant effect of time or interaction between time and group was found for the latencies for the N100, P300, or N400, components. In contrast, prior studies have observed a significant increase in P300 latency in addition to the reduced amplitude replicated in the present study (Morris et al., 1992; Lee et al., 2003, 2004; Kusztor et al., 2019; Zhang et al., 2019). Possibly our current findings are limited due to small sample size, such that an effect of sleep deprivation on latency may have been detected with larger groups.

While the present study largely replicates prior findings, it is an important first demonstration of cognitive changes related to sleep deprivation using a readily deployable and accessible clinical tool for evaluation at the point-of-care. By highlighting the practical advantages of brain vital sign monitoring of situational cognitive impairment, it is possible to begin addressing critical situations in which sleep deprivation is a factor (e.g., pilots). Portable and accessible brain vital sign evaluation enables deployment to environments outside of the traditional EEG laboratory. Furthermore, the low-cost of EEG relative to other brain imaging technologies makes this approach increasingly suitable for point-of-care evaluation and distributed clinical trials where ongoing monitoring of cognitive function is required.

### 4.2 Limitations

Some caveats should be considered: Firstly, the sample size (*N* = 30) limited the group comparison (*n* = 15 per group), and a larger sample size may have positively impacted the analysis, particularly regarding the ability to detect a time x group interaction regarding the N400, as well as analysis of ERP latency. Secondly, all participants were required to be healthy with similar chronotypes and regular sleeping patterns so these results may not be relevant to groups with clinical conditions that affect sleep and/or people with different sleeping behaviors (i.e., shift workers).

## 5 Conclusion

The cognitive effects of sleep deprivation are observed in brain vitals signs. Significant differences were observed in a group of sleep deprived participants relative to controls who experienced a typical sleeping routine. Brain vital signs can be a useful tool in researching changes in cognition in control-intervention study designs, as well as potentially in the early and sensitive detection of cognitive impairment. Ongoing work is evaluating the effect of interventions designed to enhance cognitive performance.

## Data availability statement

The datasets presented in this article are not readily available because the datasets generated and/or analyzed during the current study are not currently publicly available due to intellectual property considerations. Requests to access the datasets should be directed to RD’A, ryan@healthtechconnex.com.

## Ethics statement

The studies involving humans were approved by the Advarra Canadian Research Ethics Board. The studies were conducted in accordance with the local legislation and institutional requirements. The participants provided their written informed consent to participate in this study.

## Author contributions

KJ: Formal analysis, Methodology, Writing – original draft, Writing – review and editing, Project administration, Conceptualization. TF: Writing – original draft, Writing – review and editing, Formal analysis, Visualization. SF: Conceptualization, Methodology, Writing – original draft, Writing – review and editing. GP: Conceptualization, Methodology, Writing – review and editing. SB: Conceptualization, Methodology, Writing – review and editing. BL: Conceptualization, Methodology, Writing – review and editing. JV: Methodology, Supervision, Writing – review and editing. RD’A: Conceptualization, Methodology, Supervision, Writing – original draft, Writing – review and editing.
