# Supplementary material for: Brain vital sign monitoring of sleep deprivation detects situational cognitive impairment
Source: Front Hum Neurosci. 2024 Apr 2;18:1358551. doi: 10.3389/fnhum.2024.1358551 (PMC11018923; doi:10.3389/fnhum.2024.1358551)
Supplement: Supplementary file 1 [file Table_1.DOCX]

**Supplemental Material**

**Participant Eligibility Criteria**

***Inclusion Criteria:***

1. Any sex, between the ages of 19 and 60.

2. Able to understand the informed consent form, study procedures and willing to participate in study

3. Able to perform the testing required by the study.

4. Able to remain seated for 10 minutes

5. In good health with no history of clinically relevant neurological illness, acute disease or conditions or injury in the last 5 years.

6. A score of ≤ 11 on the Pittsburgh Sleep Quality Index (PSQI)

7. A score between 31 and 69 on the Morningness-Eveningness Questionnaire (MEQ)

8. Coffee consumption of < 5 cups per day.

9. Alcohol consumption of <15 units per week.

10. Self-described regular sleep pattern for the last 2 weeks.

11. Willing to consume caffeine in coffee form

12. Follow regular daily routine 24 hours prior to Baseline and between Baseline 1 and Baseline 2 study visits (i.e. sleep time, caffeine consumption etc.)

***Exclusion Criteria:***

1. Alcohol or CBD or THC consumption 24 hours prior to baseline and during the study

2. Currently and regularly taking sleep medications or supplements or medications that effect sleep

3. Any health condition (e.g. chronic fatigue) that would prevent the subject from completing the required testing.

4. Undergoing chemotherapy or any form of intensive long-term therapy.

5. Recent (3 months) injury or other acute condition that required treatment with pain killers or analgesics.

6. History of chronic pain or chronic headache disorders, including migraines.

7. History of TBI or condition that affects the brain or CNS.

8. Currently diagnosed with major psychiatric disorders (schizophrenia, bipolar, depression, generalized anxiety disorder)

9. Diagnosed with any memory disorders.

10. Currently diagnosed with any sleeping disorders (e.g. sleep apnea, hypersomnia, insomnia, parasomnia etc.)

11. Recent (in the last 6 months) history of alcohol or substance misuse.

12. Travel across time zones in the last 2 weeks.

13. Late night or evening shift work in the last 2 weeks.

14. Vaccination for COVID-19 within the last 72 hours prior to baseline.

15. Currently experiencing Covid-19 symptoms, including: fever or chills, cough, tiredness/fatigue, headache, sore throat, muscle or body aches, new loss of taste or smell, congestion or runny nose, nausea or vomiting, diarrhea, difficulty breathing or shortness of breath, or chest pain.)

16. If female and of child-bearing potential: pregnant, suspected or planning to become pregnant or breast-feeding

17. Contraindications for the NeuroCatch Platform:

17.1. Requires use of hearing aids or a cochlear implant

17.2. Diagnosed with tinnitus that is currently active

17.3. Temporary damage to hearing (e.g. punctured ear drum).

17.4. Implanted pacemaker or implanted electrical stimulators

17.5. Metal or plastic implants in the skull, excluding dental/facial implants.

17.6. Exposed to an investigational drug or device 30 days prior to start in this study, or concurrent or planned use of investigational drug or device while enrolled in this study*

17.7. Not proficient in the English language

17.8. Diagnosed epilepsy or history of seizures

17.9. If female and of child-bearing potential: pregnant, suspected or planning to become pregnant or breast-feeding

17.10. Unhealthy scalp (apparent open wounds and/or bruised or weakened skin)

17.11. Allergy to EEG gel
